# 2-Amino-5-chloro­pyridinium 4-hydroxy­benzoate

**DOI:** 10.1107/S1600536810004265

**Published:** 2010-02-06

**Authors:** Madhukar Hemamalini, Hoong-Kun Fun

**Affiliations:** a X-ray Crystallography Unit, School of Physics, Universiti Sains Malaysia, 11800 USM, Penang, Malaysia

## Abstract

In the title salt, C_5_H_6_ClN_2_^+^·C_7_H_5_O_3_^−^, the carboxyl­ate mean plane of the 4-hydroxy­benzoate anion is twisted by 7.16 (9)° from the attached ring. In the crystal structure, the cations and anions are linked *via* O—H⋯O and N—H⋯O hydrogen bonds, as well as C—H⋯O contacts, forming a three-dimensional network. In addition, weak π–π inter­actions involving the benzene and pyridinium rings, with centroid-to-centroid distances of 3.8941 (9) Å, are observed.

## Related literature

For background to the chemistry of substituted pyridines, see: Pozharski *et al.* (1997 ▶); Katritzky *et al.* (1996 ▶). For related structures, see: Pourayoubi *et al.* (2007 ▶); Akriche & Rzaigui (2005 ▶); Janczak & Perpétuo (2009 ▶). For details of hydrogen bonding, see: Jeffrey & Saenger (1991 ▶); Jeffrey (1997 ▶); Scheiner (1997 ▶). For hydrogen-bond motifs, see: Bernstein *et al.* (1995 ▶). For the stability of the temperature controller used in the data collection, see: Cosier & Glazer (1986 ▶).
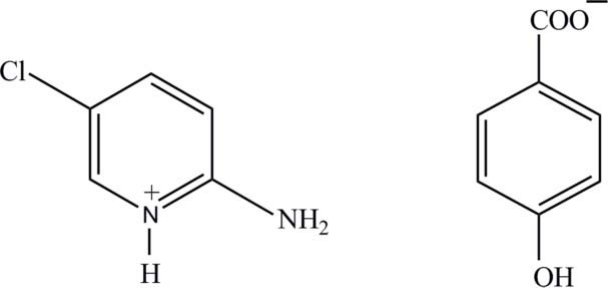


## Experimental

### 

#### Crystal data

C_5_H_6_ClN_2_^+^·C_7_H_5_O_3_^−^
*M* *_r_* = 266.68
Monoclinic, 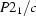

*a* = 10.0893 (3) Å
*b* = 11.7612 (4) Å
*c* = 11.6634 (3) Å
β = 116.113 (2)°
*V* = 1242.74 (6) Å^3^
*Z* = 4
Mo *K*α radiation
μ = 0.31 mm^−1^
*T* = 100 K
0.69 × 0.20 × 0.14 mm

#### Data collection

Bruker SMART APEXII CCD area-detector diffractometer
Absorption correction: multi-scan (*SADABS*; Bruker, 2009 ▶) *T*_min_ = 0.814, *T*_max_ = 0.958
12446 measured reflections
3630 independent reflections
2663 reflections with *I* > 2σ(*I*)
*R*_int_ = 0.020

#### Refinement

*R*[*F* ^2^ > 2σ(*F* ^2^)] = 0.040
*wR*(*F* ^2^) = 0.110
*S* = 1.04
3630 reflections
207 parameters
All H-atom parameters refined
Δρ_max_ = 0.22 e Å^−3^
Δρ_min_ = −0.26 e Å^−3^

### 

Data collection: *APEX2* (Bruker, 2009 ▶); cell refinement: *SAINT* (Bruker, 2009 ▶); data reduction: *SAINT*; program(s) used to solve structure: *SHELXTL* (Sheldrick, 2008 ▶); program(s) used to refine structure: *SHELXTL*; molecular graphics: *SHELXTL*; software used to prepare material for publication: *SHELXTL* and *PLATON* (Spek, 2009 ▶).

## Supplementary Material

Crystal structure: contains datablocks global, I. DOI: 10.1107/S1600536810004265/tk2625sup1.cif

Structure factors: contains datablocks I. DOI: 10.1107/S1600536810004265/tk2625Isup2.hkl

Additional supplementary materials:  crystallographic information; 3D view; checkCIF report

## Figures and Tables

**Table 1:** Hydrogen-bond geometry (Å, °)

*D*—H⋯*A*	*D*—H	H⋯*A*	*D*⋯*A*	*D*—H⋯*A*
O1—H1*O*1⋯O3^i^	0.84 (2)	1.91 (2)	2.7132 (15)	160 (2)
N1—H1*N*1⋯O2^ii^	0.99 (2)	1.66 (2)	2.6320 (18)	169.2 (18)
N2—H1*N*2⋯O2^iii^	0.857 (19)	2.051 (19)	2.8972 (18)	169 (2)
N2—H2*N*2⋯O3^ii^	0.92 (2)	1.93 (2)	2.825 (2)	167 (2)
C3—H3*A*⋯O3	0.95 (2)	2.488 (19)	3.181 (2)	129.5 (14)

Symmetry codes: (i) 
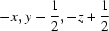
; (ii) 
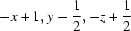
; (iii) 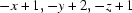.

**Fig. 1.**
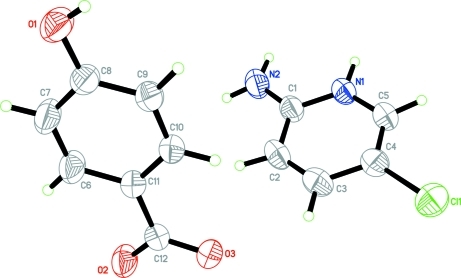
The asymmetric unit of the title compound. Displacement ellipsoids are drawn at the 50% probability level.

**Fig. 2.**
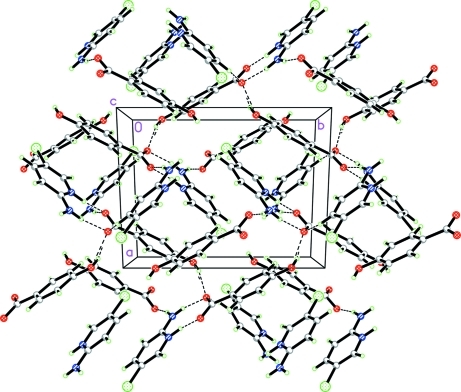
The crystal packing of the title compound, showing hydrogen-bonded (dashed lines) networks.

## supplementary crystallographic information

### Comment

Pyridine and its derivatives play an important role in heterocyclic chemistry (Pozharski *et al.*, 1997; Katritzky *et al.*, 1996). Pyridine and its substituted derivatives are often involved in hydrogen-bond interactions (Jeffrey & Saenger, 1991; Jeffrey, 1997; Scheiner, 1997). The crystal structures of 2-amino-5-chloropyridine (Pourayoubi *et al.*, 2007), 2-amino-5-chloropyridinium trichloroacetate (Janczak & Perpétuo, 2009) and bis (2-amino-5-chloropyridinium) dihydrogendiphosphate (Akriche & Rzaigui, 2005) have been reported. Since our aim is to study some interesting hydrogen-bonding interactions, the crystal structure of the title salt is presented here.

The asymmetric unit (Fig. 1) contains a 2-amino-5-chloropyridinium cation and a 4-hydroxybenzoate anion. The proton transfer from the carboxylic acid to atom N1 of 2-amino-5-chloropyridine resulted in the widening of C1—N1—C5 angle of the pyridinium ring to 122.24 (12)°, compared to the corresponding angle of 118.1 (12)° in neutral 2-amino-5-chloropyridine (Pourayoubi *et al.*, 2007). The 2-amino-5-chloropyridinium cation is essentially planar, with a maximum deviation of 0.012 (2) Å for atom C1. In the 4-hydroxybenzoate anion, the carboxylate group is twisted slightly from the attached ring; the dihedral angle between the C6–C11 and O2/O3/C11/C12 planes is 7.16 (9) °.

In the crystal packing (Fig. 2), the protonated N1 atom and the N2-amino group is hydrogen-bonded to the carboxylate oxygen atoms (O2 and O3) via a pair of N—H···O hydrogen bonds forming a *R*_2_^2^(8) ring motif (Bernstein *et al.*, 1995). The hydroxyl hydrogen atom is also hydrogen-bonded to the carboxylate oxygen atom through an O—H···O hydrogen bond. The packing is further stabilized by weak C—H···O contacts, Table 1, and π···π interactions involving the benzene (centroid Cg1) and pyridinium (centroid Cg2) rings, with Cg1–Cg2 = 3.8941 (9) Å [symmetry code: x, 3/2 - y, 1/2 + z].

### Experimental

A hot methanol solution (20 ml) of 2-amino-5-chloropyridine (65 mg, Aldrich) and 4-hydroxybenzoic acid (69 mg, Merck) were mixed and warmed over a heating magnetic stirrer for a few minutes. The resulting solution was allowed to cool slowly at room temperature and crystals of the title compound appeared after a few days.

### Refinement

All the H atoms were located in a difference Fourier map and allowed to refine freely [N—H = 0.858 (19)–0.99 (2) Å, O—H = 0.83 (2) Å, C—H = 0.925 (19)–0.965 (16) Å].

### Crystal data

**Table:**

C_5_H_6_ClN_2_^+^·C_7_H_5_O_3_^−^	*F*(000) = 552
*M**_r_* = 266.68	*D*_x_ = 1.425 Mg m^−^^3^
Monoclinic, *P*2_1_/*c*	Mo *K*α radiation, λ = 0.71073 Å
Hall symbol: -P 2ybc	Cell parameters from 4875 reflections
*a* = 10.0893 (3) Å	θ = 2.3–29.9°
*b* = 11.7612 (4) Å	µ = 0.31 mm^−^^1^
*c* = 11.6634 (3) Å	*T* = 100 K
β = 116.113 (2)°	Block, colourless
*V* = 1242.74 (6) Å^3^	0.69 × 0.20 × 0.14 mm
*Z* = 4	

### Data collection

**Table:**

Bruker SMART APEXII CCD area-detector diffractometer	3630 independent reflections
Radiation source: fine-focus sealed tube	2663 reflections with *I* > 2σ(*I*)
graphite	*R*_int_ = 0.020
φ and ω scans	θ_max_ = 30.1°, θ_min_ = 2.3°
Absorption correction: multi-scan (*SADABS*; Bruker, 2009)	*h* = −13→14
*T*_min_ = 0.814, *T*_max_ = 0.958	*k* = −16→16
12446 measured reflections	*l* = −16→16

### Refinement

**Table:**

Refinement on *F*^2^	Primary atom site location: structure-invariant direct methods
Least-squares matrix: full	Secondary atom site location: difference Fourier map
*R*[*F*^2^ > 2σ(*F*^2^)] = 0.040	Hydrogen site location: inferred from neighbouring sites
*wR*(*F*^2^) = 0.110	All H-atom parameters refined
*S* = 1.04	*w* = 1/[σ^2^(*F*_o_^2^) + (0.0478*P*)^2^ + 0.2067*P*] where *P* = (*F*_o_^2^ + 2*F*_c_^2^)/3
3630 reflections	(Δ/σ)_max_ < 0.001
207 parameters	Δρ_max_ = 0.22 e Å^−^^3^
0 restraints	Δρ_min_ = −0.26 e Å^−^^3^

### Special details

**Table:**

Experimental. The crystal was placed in the cold stream of an Oxford Cryosystems Cobra open-flow nitrogen cryostat (Cosier & Glazer, 1986) operating at 100.0 (1) k.
Geometry. All s.u.'s (except the s.u. in the dihedral angle between two l.s. planes) are estimated using the full covariance matrix. The cell s.u.'s are taken into account individually in the estimation of s.u.'s in distances, angles and torsion angles; correlations between s.u.'s in cell parameters are only used when they are defined by crystal symmetry. An approximate (isotropic) treatment of cell s.u.'s is used for estimating s.u.'s involving l.s. planes.
Refinement. Refinement of F^2^ against ALL reflections. The weighted R-factor wR and goodness of fit S are based on F^2^, conventional R-factors R are based on F, with F set to zero for negative F^2^. The threshold expression of F^2^ > 2σ(F^2^) is used only for calculating R-factors(gt) etc. and is not relevant to the choice of reflections for refinement. R-factors based on F^2^ are statistically about twice as large as those based on F, and R- factors based on ALL data will be even larger.

### Fractional atomic coordinates and isotropic or equivalent isotropic displacement parameters (Å^2^)

**Table:**

	*x*	*y*	*z*	*U*_iso_*/*U*_eq_	
Cl1	0.21064 (4)	0.98776 (4)	−0.17272 (4)	0.06226 (15)	
N1	0.51321 (13)	0.78671 (10)	0.07645 (11)	0.0444 (3)	
N2	0.63089 (16)	0.77385 (14)	0.29586 (13)	0.0574 (3)	
C1	0.53134 (15)	0.82441 (12)	0.19187 (13)	0.0439 (3)	
C2	0.44186 (16)	0.91497 (14)	0.19530 (14)	0.0510 (4)	
C3	0.34358 (17)	0.96337 (14)	0.08507 (15)	0.0512 (4)	
C4	0.33162 (14)	0.92372 (13)	−0.03259 (13)	0.0454 (3)	
C5	0.41534 (15)	0.83516 (13)	−0.03444 (13)	0.0454 (3)	
O1	0.00092 (13)	0.66336 (10)	0.45630 (11)	0.0579 (3)	
O2	0.34526 (13)	1.11961 (9)	0.47186 (9)	0.0567 (3)	
O3	0.22698 (11)	1.09176 (8)	0.26301 (8)	0.0463 (2)	
C6	0.21056 (18)	0.92410 (14)	0.51840 (13)	0.0531 (4)	
C7	0.14592 (19)	0.82697 (15)	0.53591 (13)	0.0563 (4)	
C8	0.06192 (15)	0.75826 (12)	0.43312 (13)	0.0438 (3)	
C9	0.04555 (15)	0.78713 (12)	0.31209 (12)	0.0426 (3)	
C10	0.11107 (14)	0.88452 (12)	0.29507 (12)	0.0397 (3)	
C11	0.19355 (14)	0.95522 (12)	0.39715 (11)	0.0392 (3)	
C12	0.25875 (14)	1.06243 (12)	0.37582 (12)	0.0398 (3)	
H2A	0.4538 (17)	0.9382 (14)	0.2769 (16)	0.059 (5)*	
H3A	0.283 (2)	1.0251 (15)	0.0866 (17)	0.064 (5)*	
H5A	0.4139 (17)	0.8021 (14)	−0.1096 (16)	0.060 (5)*	
H6A	0.2676 (19)	0.9721 (15)	0.5889 (17)	0.065 (5)*	
H7A	0.1571 (19)	0.8087 (16)	0.6168 (18)	0.068 (5)*	
H9A	−0.0099 (17)	0.7369 (13)	0.2417 (15)	0.054 (4)*	
H10A	0.1007 (16)	0.9043 (13)	0.2125 (15)	0.048 (4)*	
H1O1	−0.053 (2)	0.6331 (17)	0.386 (2)	0.073 (6)*	
H1N1	0.575 (2)	0.7250 (16)	0.0689 (18)	0.076 (6)*	
H1N2	0.6509 (19)	0.8050 (16)	0.3681 (18)	0.062 (5)*	
H2N2	0.689 (2)	0.7177 (19)	0.2877 (19)	0.080 (6)*	

### Atomic displacement parameters (Å^2^)

**Table:**

	*U*^11^	*U*^22^	*U*^33^	*U*^12^	*U*^13^	*U*^23^
Cl1	0.0607 (2)	0.0623 (3)	0.0572 (2)	0.00938 (18)	0.01990 (18)	0.00670 (19)
N1	0.0523 (6)	0.0433 (6)	0.0410 (6)	0.0039 (5)	0.0235 (5)	−0.0053 (5)
N2	0.0729 (8)	0.0590 (9)	0.0403 (6)	0.0110 (7)	0.0249 (6)	−0.0023 (6)
C1	0.0515 (7)	0.0438 (7)	0.0425 (6)	−0.0042 (6)	0.0262 (6)	−0.0069 (6)
C2	0.0587 (8)	0.0536 (9)	0.0476 (7)	0.0000 (7)	0.0297 (6)	−0.0132 (7)
C3	0.0528 (7)	0.0479 (9)	0.0585 (8)	0.0037 (7)	0.0297 (7)	−0.0092 (7)
C4	0.0441 (6)	0.0451 (8)	0.0475 (7)	−0.0022 (6)	0.0206 (5)	−0.0043 (6)
C5	0.0504 (7)	0.0467 (8)	0.0416 (7)	0.0000 (6)	0.0226 (6)	−0.0071 (6)
O1	0.0716 (7)	0.0539 (7)	0.0450 (5)	−0.0210 (6)	0.0228 (5)	0.0014 (5)
O2	0.0799 (7)	0.0531 (6)	0.0401 (5)	−0.0245 (5)	0.0291 (5)	−0.0082 (5)
O3	0.0597 (5)	0.0440 (5)	0.0370 (4)	0.0000 (4)	0.0229 (4)	0.0042 (4)
C6	0.0709 (9)	0.0536 (9)	0.0333 (6)	−0.0166 (8)	0.0217 (6)	−0.0052 (6)
C7	0.0763 (10)	0.0591 (10)	0.0347 (6)	−0.0180 (8)	0.0256 (7)	0.0003 (7)
C8	0.0486 (7)	0.0423 (7)	0.0406 (6)	−0.0039 (6)	0.0197 (5)	0.0024 (6)
C9	0.0485 (6)	0.0418 (7)	0.0345 (6)	−0.0032 (6)	0.0155 (5)	−0.0028 (6)
C10	0.0466 (6)	0.0403 (7)	0.0329 (6)	0.0027 (6)	0.0181 (5)	0.0014 (5)
C11	0.0459 (6)	0.0384 (7)	0.0348 (6)	0.0000 (5)	0.0192 (5)	−0.0001 (5)
C12	0.0480 (6)	0.0380 (7)	0.0372 (6)	0.0018 (6)	0.0224 (5)	−0.0011 (5)

### Geometric parameters (Å, °)

**Table:**

Cl1—C4	1.7241 (15)	O1—H1O1	0.83 (2)
N1—C1	1.3511 (17)	O2—C12	1.2682 (15)
N1—C5	1.3595 (18)	O3—C12	1.2577 (15)
N1—H1N1	0.99 (2)	C6—C7	1.375 (2)
N2—C1	1.3271 (19)	C6—C11	1.3963 (18)
N2—H1N2	0.858 (19)	C6—H6A	0.954 (18)
N2—H2N2	0.91 (2)	C7—C8	1.384 (2)
C1—C2	1.408 (2)	C7—H7A	0.925 (19)
C2—C3	1.356 (2)	C8—C9	1.3892 (19)
C2—H2A	0.946 (16)	C9—C10	1.3798 (19)
C3—C4	1.403 (2)	C9—H9A	0.965 (16)
C3—H3A	0.953 (18)	C10—C11	1.3889 (18)
C4—C5	1.347 (2)	C10—H10A	0.950 (15)
C5—H5A	0.953 (17)	C11—C12	1.4929 (19)
O1—C8	1.3581 (17)		
			
C1—N1—C5	122.24 (12)	C7—C6—C11	120.90 (13)
C1—N1—H1N1	121.1 (11)	C7—C6—H6A	120.6 (11)
C5—N1—H1N1	116.5 (11)	C11—C6—H6A	118.5 (11)
C1—N2—H1N2	117.6 (12)	C6—C7—C8	120.42 (13)
C1—N2—H2N2	119.3 (13)	C6—C7—H7A	119.5 (11)
H1N2—N2—H2N2	121.8 (18)	C8—C7—H7A	120.1 (11)
N2—C1—N1	118.66 (13)	O1—C8—C7	117.77 (12)
N2—C1—C2	123.35 (13)	O1—C8—C9	122.79 (12)
N1—C1—C2	117.98 (13)	C7—C8—C9	119.43 (13)
C3—C2—C1	120.07 (13)	C10—C9—C8	119.92 (12)
C3—C2—H2A	123.3 (10)	C10—C9—H9A	121.4 (10)
C1—C2—H2A	116.6 (10)	C8—C9—H9A	118.7 (10)
C2—C3—C4	120.00 (14)	C9—C10—C11	121.19 (12)
C2—C3—H3A	120.6 (11)	C9—C10—H10A	120.2 (9)
C4—C3—H3A	119.4 (11)	C11—C10—H10A	118.7 (9)
C5—C4—C3	119.20 (13)	C10—C11—C6	118.13 (13)
C5—C4—Cl1	120.66 (11)	C10—C11—C12	120.30 (11)
C3—C4—Cl1	120.14 (11)	C6—C11—C12	121.56 (12)
C4—C5—N1	120.45 (13)	O3—C12—O2	122.55 (12)
C4—C5—H5A	125.1 (10)	O3—C12—C11	118.55 (11)
N1—C5—H5A	114.5 (10)	O2—C12—C11	118.89 (11)
C8—O1—H1O1	108.3 (14)		
			
C5—N1—C1—N2	−178.89 (13)	C6—C7—C8—C9	1.2 (2)
C5—N1—C1—C2	1.8 (2)	O1—C8—C9—C10	−179.65 (13)
N2—C1—C2—C3	179.25 (15)	C7—C8—C9—C10	−0.9 (2)
N1—C1—C2—C3	−1.5 (2)	C8—C9—C10—C11	−0.2 (2)
C1—C2—C3—C4	−0.4 (2)	C9—C10—C11—C6	1.0 (2)
C2—C3—C4—C5	2.1 (2)	C9—C10—C11—C12	−177.69 (12)
C2—C3—C4—Cl1	−178.10 (12)	C7—C6—C11—C10	−0.8 (2)
C3—C4—C5—N1	−1.8 (2)	C7—C6—C11—C12	177.90 (15)
Cl1—C4—C5—N1	178.38 (11)	C10—C11—C12—O3	5.95 (19)
C1—N1—C5—C4	−0.2 (2)	C6—C11—C12—O3	−172.73 (13)
C11—C6—C7—C8	−0.3 (3)	C10—C11—C12—O2	−173.19 (12)
C6—C7—C8—O1	179.94 (15)	C6—C11—C12—O2	8.1 (2)

### Hydrogen-bond geometry (Å, °)

**Table:**

*D*—H···*A*	*D*—H	H···*A*	*D*···*A*	*D*—H···*A*
O1—H1O1···O3^i^	0.84 (2)	1.91 (2)	2.7132 (15)	160 (2)
N1—H1N1···O2^ii^	0.99 (2)	1.66 (2)	2.6320 (18)	169.2 (18)
N2—H1N2···O2^iii^	0.857 (19)	2.051 (19)	2.8972 (18)	169 (2)
N2—H2N2···O3^ii^	0.92 (2)	1.93 (2)	2.825 (2)	167 (2)
C3—H3A···O3	0.95 (2)	2.488 (19)	3.181 (2)	129.5 (14)

Symmetry codes: (i) −*x*, *y*−1/2, −*z*+1/2; (ii) −*x*+1, *y*−1/2, −*z*+1/2; (iii) −*x*+1, −*y*+2, −*z*+1.
